# Eukaryotic Translation Initiation Factor 5A Independently Predicts Poor Prognosis of Cholangiocarcinoma Patients and Regulates the Ferroptosis and Mitochondrial Apoptosis

**DOI:** 10.1155/2022/4250531

**Published:** 2022-07-15

**Authors:** Ping Wan, Taiyuan Li, Longfei Zhou, Jun Zhang, Xuefeng Rao

**Affiliations:** ^1^Department of General Surgery, Jiangxi Provincial People's Hospital, Nanchang 330006, Jiangxi Province, China; ^2^Department of General Surgery, First Affiliated Hospital of Nanchang University, Nanchang 330006, Jiangxi Province, China

## Abstract

Cholangiocarcinoma (CCA) is a hepatobiliary carcinoma characterized by the differentiation of bile duct cells, and the patients with CCA often have a poor prognosis. Eukaryotic translation initiation factor 5A (eIF5A) is reported to have multiple biological activities. Targeted activation of ferroptosis may be a therapeutic strategy for cancer. Nevertheless, the effects of eIF5A and ferroptosis on CCA are still elucidated. Our study explored the effects of eIF5A in CCA, and the mechanisms also are studied. In this paper, TCGA database analysis suggested that eIF5A was upregulated in CCA, and high expression of eIF5A might predict a poor prognosis. Moreover, FANCD2, SLC7A11, and HSPB1 were significantly overexpressed in CCA. The results indicated that eIF5A was overexpressed in CCA tissues and cells. Further experiments demonstrated that eIF5A silencing decreased CCA cell activity and enhanced ferroptosis and mitochondrial apoptosis. In addition, upregulation of eIF5A showed the opposite effect on CCA cells compared with downregulation of eIF5A. Finally, the silencing of eIF5A could restrain the growth of xenografted tumors and promote ferroptosis. Overall, eIF5A enlarged CCA cell activity and attenuated ferroptosis and mitochondrial apoptosis. The results suggested that assessment of eIF5A might provide help for the diagnosis and treatment of CCA.

## 1. Introduction

Cholangiocarcinoma (CCA) is a rare malignancy belonging to hepatobiliary surgery [1]. Globally, hepatobiliary malignancies account for 13% of cancer-related deaths, and 10%–20% of these are attributable to CCA [2]. Incidence rate and mortality rate of CCA show a markedly increasing trend over recent years [3]. At present, the risk factors of CCA are complicated, including enteritis, drinking, smoking, diabetes, and gallstones [4, 5]. CCA develops from epithelial cells of bile duct [6]. Due to the characteristics of hidden onset, rapid developing, and difficult early diagnosis, most patients with CCA exhibit no symptoms and are frequently misdiagnosed [7, 8]. Therefore, we urgently need to find new molecular markers to effectively evaluate the progression of CCA, so as to facilitate the treatment of CCA.

Accumulation of reactive oxygen species (ROS), inhibition of glutathione (GSH) activity, and mitochondria injury are main characteristics of ferroptosis [9, 10]. Ferroptosis participates in regulating tumorigenesis [11, 12]. Nedd4 silencing could promote ferroptosis by inhibiting the degradation of VDAC2/3, so as to resist the drug resistance of ferroptosis-activator erastin in cancer cells [13]. Stearoyl-CoA destruction 1 (SCD1) amplified gastric cancer cell growth, migration, and antiferroptosis by regulating cancer stemness and cell cycle in gastric cancer patients [14]. Activating of ferroptosis showed great potential for cancer treatment [15]. It is crucial for cancer treatment to study the underlying molecular mechanism and signal pathway of ferroptosis. Therefore, ferroptosis was explored in CCA in this paper.

Eukaryotic translation initiation factor 5A (eIF5A) is a class of highly conserved proteins in eukaryotic cells [16]. eIF5A plays important roles in cell differentiation, cell proliferation, cell death, and nuclear transport [16–18]. eIF5A was upregulated in multiple malignancies and closely related to poor prognosis [19]. Inhibition of eIF5A/sHH signaling pathway attenuated the growth of pancreatic cancer (PC) cells and increased gemcitabine sensitivity for PC [17]. In gastric cancer, high expression of eIF5A-2 was relative to a poor prognosis [20]. Knockdown of eIF5A or treatment with DHPs inhibitor (GC7) could inhibit the hypusination of eIF5A, which restrained the growth of colorectal cancer cells through arresting the synthesis of MYC protein [16]. However, the roles of eIF5A in CCA and ferroptosis have not yet been studied.

In this paper, bioinformatics analysis showed that overexpressed eIF5A was found in CCA tissues compared with normal tissues. Moreover, our clinical data similarly indicated that eIF5A was overexpressed in CCA, and expression of eIF5A was negative correlation with overall survival and positively correlated with pT_stage and pTNM_stage. Moreover, we evaluated the effect of eIF5A on ferroptosis of CCA cells and the corresponding mechanism in vitro and in vivo. The results suggested that eIF5A increased cell viability and restrained ferroptosis and mitochondrial apoptosis in CCA. eIF5A might be a potential target gene for the treatment of CCA.

## 2. Materials and Methods

### 2.1. Data Collection

Clinical data from patients with cholangiocarcinoma were obtained from the Cancer Genome Atlas (TCGA) (http://gdc-portal.nci.nih.gov/) [21]. Samples with incomplete information were removed in advance before analysis. Clinicopathological data included survival status, age, gender, race, pT, pN, pM, pTNM stage, and tumor type. Expression comparisons of ferroptosis-related genes in 36 CCA tissues and 9 normal samples from TCGA were analyzed with the following cut-off criteria: fold change (FC) > 1 and *P* < 0.05, and Top 12 genes of upregulation and downregulation were displayed by heat map.

### 2.2. Tumor IMmune Estimation Resource (TIMER) Database

TIMER (https://cistrome.shinyapps.io/timer/) is often used for analyzing the relation between the infiltration of immunocyte and the clinical impact [22]. The effect of eIF5A expression on the degree of infiltration of immune cells in CCA was evaluated.

### 2.3. Tissue Samples

Tumor samples were collected from patients with CCA treated in Affiliated People's Hospital of Nanchang University Hospital. Patients with CCA were pathologically confirmed by authoritative experts and did not receive chemotherapy and radiotherapy before operation. The patients signed the informed consent form, which was signed by all participants. Moreover, this experiment was with the consent of the ethics committee of the Affiliated People's Hospital of Nanchang University.

### 2.4. Cell Culture and Transfection

CCA cells (HuCCT1, TFK-1, KKU-452, KKU-100, and QBC939) and human intrahepatic biliary epithelial cells (HIBEpiC) were purchased from ATCC. The DMEM medium (Invitrogen, USA) containing 10% fetal bovine serum (Gibco, USA) was used for cells. The culture conditions were 37°C and 5% CO_2_ in an incubator.

The cells were inoculated into 6-well plates according to the density of 2.5 × 10^5^/well. sh-eIF5A^#^1 (5′-GCATTACGTAAGAATGGCTTT-3′), sh-eIF5A^#^2 (5′-GCATTCAAGATGGTTACCTTT-3′), sh-eIF5A^#^3 (5′-GCCATGTAAGATCGTCGAGAT-3′), shRNA-NC (5′-TTCTCCGAACGTGTCACGT-3′), pcDNA, and pcDNA-eIF5A were obtained from GenePharma (Shanghai, China). As previous studies [23], after overnight of culture, the serum-free medium was replaced, and then transfection was carried out by lipofectamine 3000 (Invitrogen). After 6 h of culture, medium was replaced; then the cells were cultured for 24 h. Following that, subsequent experiments were performed.

### 2.5. CCK8 Assay

The transfected cells (5 × 10^3^/well) were seeded into 96-well plates. After 24 h of culture, each well was added with 10 *μ*l CCK8 reagent (Beyotime, China). After 2 h, the absorbance at 450 nm was measured through a microplate reader (Bio-Rad).

### 2.6. Reverse Transcription-Polymerase Chain Reaction (RT-PCR)

Total RNA was isolated with Trizol reagent (Invitrogen). Single stranded cDNA was obtained through 1st Strand cDNA Synthesis kit (H6110 A, Takara, Japan). Then, RT-PCR experiment was carried out according to the instructions of SYBR@ Premix Ex TaqTM II kit (HRR081 B, Takara, Japan) on ABI7900HT system (Applied Biosystems). The reaction conditions were 95°C for 3 min and 35 cycles (94°C for 30 s, 58°C for 30 s, and 72°C for 50 s). The results were analyzed according to 2^−ΔΔ CT^ method. GAPDH is used as internal parameter. The primers were as follows: eIF5A1 forward: 5′-GACTTCCAGCTGATTGGCATCCAG, reverse: 5′-GCGGGCCTTATTTTGCCATGGCCTTGATTG; GAPDH forward: 5′-ATGGGGAAGGTGAAGGTCG-3′, reverse: 5′-TAAAAGCAGCCCTGGTGACC-3′.

### 2.7. Western Blot

Tissue homogenates and cells were treated with RIPA lysates (Beyotime, China), and supernatants containing proteins were collected by centrifugation. Protein content was assessed by BCA detection kit (EMD Millipore). SDS-PAGE was performed with the same amount of protein samples in each lane, and then the isolated proteins were electrotransferred to PVDF membrane (Millipore). 5% skimmed milk powder was used for blocking the membranes, and then the corresponding primary antibody (eIF5A (1 : 1000, ab32443, Abcam); FANCD2 (1 : 1000, ab108928, Abcam); SLC7A11 (1 : 1000, ab216876, Abcam); HSPB1 (1 : 1000, ab109376, Abcam); Bax (1 : 1000, ab53154, Abcam); Bcl-2 (1 : 1000, ab32124, Abcam); cleaved caspase-3 (1 : 1000, ab32042, Abcam); cytochrome C (1 : 1000, ab133504, Abcam); *β*-actin (1 : 1000, ab8226, Abcam)) was applied overnight at 4°C. Following that, the membranes were treated for 2 h with the corresponding secondary antibody (Goat Anti-Rabbit IgG H&L (1 : 2000, ab6721, Abcam) and Rabbit Anti-Mouse IgG H&L (1 : 2000, ab6728, Abcam)). The transfer protein on membranes was developed with electrochemiluminescence (ECL, Thermo Fisher Scientific, USA)). Grayscale of the strips was assessed by ImageJ 1.48*v* software (NIH).

### 2.8. Hoechst 33258 Staining

When the cells grew to 80%, the supernatant was discarded, and then the cells were fixed for 10 min with 4% paraformaldehyde (0.5 ml). After washing, the cells were stained for 5 min with 0.5 ml Hoechst33258 staining solution (Invitrogen). After washing, antifluorescence quenching reagent was used to seal the slide. Then the apoptotic cells were observed on the inverted fluorescence microscope (Nikon C2 Plus, Tokyo, Japan). The nucleus of the positive cells was blue.

### 2.9. Flow Cytometry

The cells were fixed for overnight at 4°C using precooled 75% ethanol. Ethanol was removed by centrifugation (1000 rpm, 5 min). Then the cells were mixed with 0.5 mg/L ethidium iodide (PI) and annexin V (Invitrogen). Within 1 h, flow cytometry was used for measurement of cell apoptosis (BD Bioscience, USA).

### 2.10. Detection of ROS

The cells (2.5 × 10^5^/well) were inoculated into 6-well plates. After 24 h, 1 ml DCFH-DA (Beyotime, China) (2 *μ*M) was added into each well and then incubated for 20 min at 37°C avoiding light. Following that, a fluorescence microscope was applied for observing the results. ImageJ 1.48*v* software (NIH) was used to analyze the results.

### 2.11. Detection of Oxidative Stress and Fe^2+^

The cells (2.5 × 10^5^/well) transfected with sh-eIF5A, shRNA-NC, pcDNA, or pcDNA-eIF5A were inoculated into a 6-well plate. After 24 h, the cells were treated for 30 min with RIPA lysate, and the supernatant was collected. Then the Fe^2+^, SOD, and MDA contents were detected through Iron assay kit (Beijing Applygen Technologies, China), SOD detection kit, and MDA detection kit (A001-3-2 and A003-1-2, Nanjing Jincheng Bioengineering Institute), respectively.

The cells transfected with sh-eIF5A, shRNA-NC, pcDNA, or pcDNA-eIF5A were treated with erastin (10 *μ*M) [24] or ferrostatin-1 (1 *μ*M) [25] for 24 h. Then the Fe^2+^ contents were detected through Iron assay kit. Ferrostatin-1 (S7243) and erastin (S7242) were purchased from Selleck (Shanghai, China).

### 2.12. Xenografted Tumors

Female nude BALB/c mice (*n* = 19, 6–8 weeks old) were purchased from Shanghai Lab, Animal Research Center. The HuCCT1 cells (5 × 10^6^ in phosphate buffer saline, 200 *µ*l) transfected with sh-eIF5A or shRNA-NC were subcutaneously inoculated in the dorsal near the right forelimb of nude mice in a sterile environment. The tumor growth was observed every day. The diameter of the xenografted tumors was assessed at the 5th day after inoculation. The nude mice with the diameter of the transplanted tumor of 3–5 mm were used in the follow-up experiment. The tumor volume was measured every 5 d after inoculation. After 30 d, the nude mice were euthanized by intraperitoneal injection of pentobarbital sodium (120 mg/kg). During the experiment, when dyspnea, diarrhea, incontinence, rapid weight loss, and loss of appetite (more than 24 h without eating and drinking) were observed, the rats should be euthanized [26]. The death of rats was determined by observing the cardiac arrest and pupil dilation [27]. Animal health and behaviour were monitored every 3 d. The tumor tissue was stripped, photographed, and weighed, and the tumor growth curve was drawn. Animal experiments were with the consent of the ethics committee of Affiliated People's Hospital of Nanchang University.

### 2.13. TUNEL Assay

The tumor tissue was fixed for 24 h with paraformaldehyde, dehydrated and embedded in paraffin, and then made into 4 *μ*m continuous sections. The sections were used for TUNEL staining, which was performed using the TUNEL system (GS0249, Biolab, Beijing). After dewaxing and hydrating, the sections were treated for 30 min with protease K (20 *μ*g/ml, 10 mM Tris/HCl, pH = 7.4–8.0) at 37°C, hatched for 60 min with 50 *μ*l TUNEL at 37°C, incubated for 30 min using 50 *μ*l AP antibody at 37°C, and treated for 20 min with BCIP/NBT at 37°C, and counterstain and seal were performed. The results were observed under an optical microscope (Olympus Corporation). The apoptotic cells with the nucleus dyed blue and black were observed. Randomly taken 5 visual fields under 400 *X* microscope were observed.

### 2.14. Hematoxylin-Eosin (HE) Staining

After dewaxing and hydration, the sections were stained with hematoxylin solution for 5 min, differentiated with 1% hydrochloric acid alcohol for 5 s, and then stained with 1% eosin solution for 1 min, and sealed with neutral resin. Following that, the results were observed under an optical microscope (Olympus Corporation).

### 2.15. Immunohistochemistry

Slices were dewaxed and hydrated. Endogenous catalase was removed by H_2_O_2_. The slices were blocked for 30 min at 37°C with 5% BSA solution (P0220, Beyotime). Following that, the sections were reacted with primary antibody [eIF5A (1 : 250, ab32443, Abcam); FANCD2 (1 : 100, ab108928, Abcam); SLC7A11 (1 : 500, ab216876, Abcam); HSPB1 (1 : 500, ab109376, Abcam)] overnight at 4°C. The sections were incubated with the secondary antibody for 30 min. Then the slides were treated for 5 min with diaminobenzidine (DAB, Beyotime, China). After restaining for 5 min with hematoxylin, the slices were dehydrated, made transparent, and finally sealed with neutral resin. The results were observed with an optical microscope (Olympus Corporation).

### 2.16. Statistical Analysis

**Figure 1:**
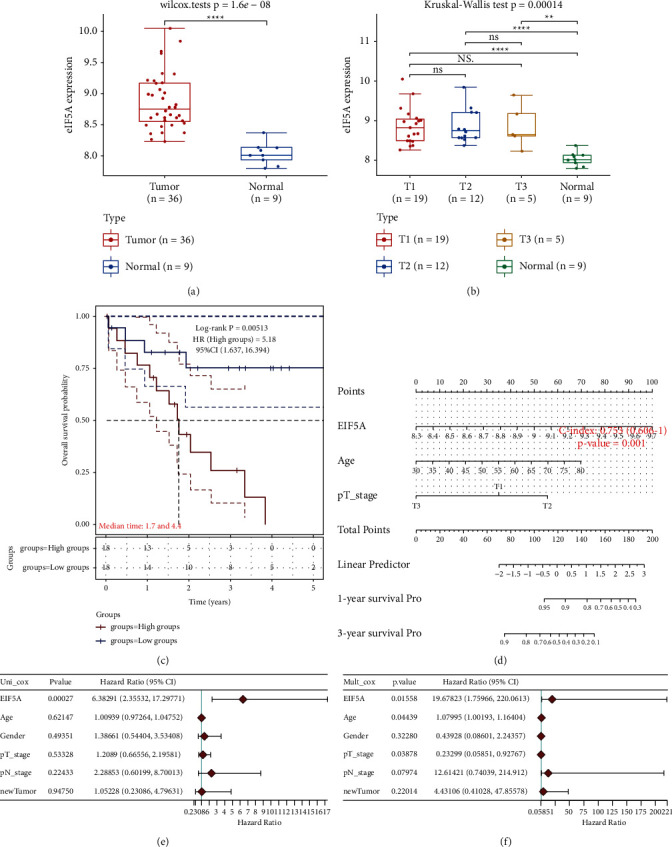
High expression of eIF5A predicted a poor prognosis. (a) Expression of eIF5A in CCA was analyzed by TCGA database. (b) Expression of eIF5A in T1, T2, and T3 stages of CCA was evaluated by TCGA database. (c) Relationship of expression of eIF5A and overall survival was analyzed by Kaplan–Meier methods. (d, e) The prognostic significance of the clinical characteristics (age, sex, pT, pN, and tumor type) and eIF5A expression were analyzed by univariate and multivariate cox proportional regression models. (f) The nomograms of 1-year and 3-year overall survival rates of patients with CCA were constructed by *R* software package “RMS” based on the analysis results of multivariate cox regression. ^*∗∗*^*p* < 0.01 vs. normal group; ^*∗∗∗∗*^*p* < 0.0001 vs. normal group.

SPSS 22 (IBM Corp.) and GraphPad 5.0 (GraphPad Software, Inc.) were used for data analysis. Wilcoxon's test was used to compare KRT15 expression between tumor tissues and normal tissues in Figure 1(a). The Kruskal–Wallis test with Dunn post hoc tests was performed to determine relationships between pT stage and KRT15 expression in Figure 1(b). Two-tailed *t*-test was used for comparison between the two groups, one-way ANOVA followed Newman–Keuls post-test was used for comparison among multiple groups, and one-way ANOVA followed Tukey's post-test was used where >4 groups were being compared. The relationship between eIF5A expression and overall survival was evaluated by Kaplan–Meier curve and log-rank test. The prognostic significance of the clinical characteristics (age, sex, pT, pN, and tumor type) and eIF5A expression were analyzed by univariate and multivariate cox proportional regression models. The nomograms of 1-year and 3-year overall survival rates of patients with CCA were constructed by *R* software package “RMS” based on the analysis results of multivariate cox regression. The *p* values, hazard ratios (HRs), and 95% confidence intervals (95% CIs) were obtained. When *p* < 0.05, the results were considered significant difference.

## 3. Results

### 3.1. Abnormal Overexpression and Potential Prognostic Value of eIF5A in CCA

The effect of the expression of eIF5A on prognosis was researched in CCA. The database information showed that eIF5A was overexpressed in CCA tissues (Figure 1(a)), and eIF5A expression in CCA tissues of T1, T2, and T3 stages patients was upregulated (Figure 1(b)). Moreover, patients with high expression of eIF5A showed shorter overall survival than the patients with low expression of eIF5A (HR = 5.18, 95% CI = 1.637–16.394, *p* < 0.01) (Figure 1(c)). Relevance of clinical characteristics (eIF5A expression, age, gender, pT stage, pN stage, and tumor type) (Table 1) and prognosis were evaluated through univariate and multivariate cox proportional regression models. The results of univariate cox analysis showed that the risk of death in the high eIF5A expression group was 6.382 times higher than that in the low eIF5A expression group (HR = 6.382, 95% CI = 2.355–17.297, *p* < 0.001) (Figure 1(e)), indicating that eIF5A expression might affect the prognosis of CCA. However, in multivariate cox analysis, eIF5A expression (HR = 19.678, 95% CI = 1.759–220.061, *p* < 0.05), age (HR = 1.079, 95% CI = 1.001–1.164, *p* < 0.05), and pT stage (HR = 0.232, 95% CI = 0.058–0.927, *p* < 0.05) might be independent risk factors for patients with CCA (Figure 1(f)). Then, based on the results of multivariate cox analysis, we established a nomogram with *R* language, which could predict the 1-year and 3-year survival rates of patients with CCA. The results showed that the concordance index (C-index) of the nomogram was 0.753 (95% CI = 0.606–1, *p* < 0.01) (Figure 1(d)). In addition, the relation of eIF5A expression and immune cell infiltration was explored through TIMER database, and the results showed that eIF5A expression has no significant correlation with the infiltration of purity cells, B cells, CD8+ T cells, CD4+ T cells, macrophages, neutrophils, and dendritic cells in ACC (Supplementary Figure 1). These results suggested that eIF5A was overexpressed, and high expression of eIF5A might be a potential prognostic indicator in CCA.

### 3.2. Abnormal Expression of Ferroptosis-Relative Genes in CCA

**Figure 2:**
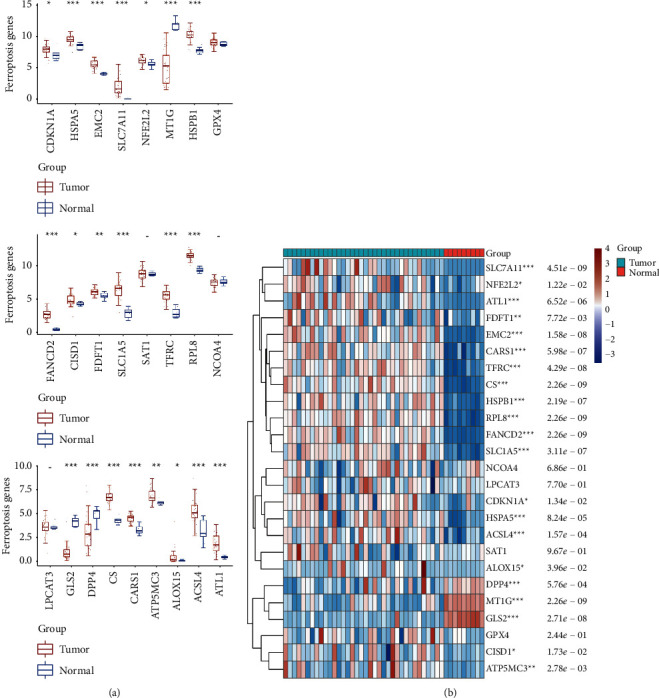
Abnormal expression of ferroptosis markers in CCA. (a) Ferroptosis markers in CCA were evaluated by TCGA database. (b) Abnormal expression of ferroptosis markers was shown in heat map. ^*∗*^*p* < 0.05 vs. normal group, ^*∗∗*^*p* < 0.01 vs. normal group, and ^*∗∗∗*^*p* < 0.001 vs. normal group.

The role of ferroptosis in CCA has rarely been studied. Ferroptosis-relative genes were screened from the database in our study. Our finding showed that abnormally expressed genes included CDKN1A, HSPA5, EMC2, SLC7A11, NFE2L2, MT1G, HSPB1, FANCD2, CISD1, FDFT1, SLC1A5, TFRC, RPL8, GLS2, DPP4, CS, CARS1, ATP5MC3, ALOX15, ACSL4, and ATL1 in CCA (Figure 2(a)). The three most significantly upregulated genes (FANCD2, SLC7A11, and HSPB1) (Figure 2(b)) shown in the heat map were selected for subsequent experimental studies.

### 3.3. eIF5A Was Upregulated in CCA Tissues and Cells and Promoted Cell Growth

**Figure 3:**
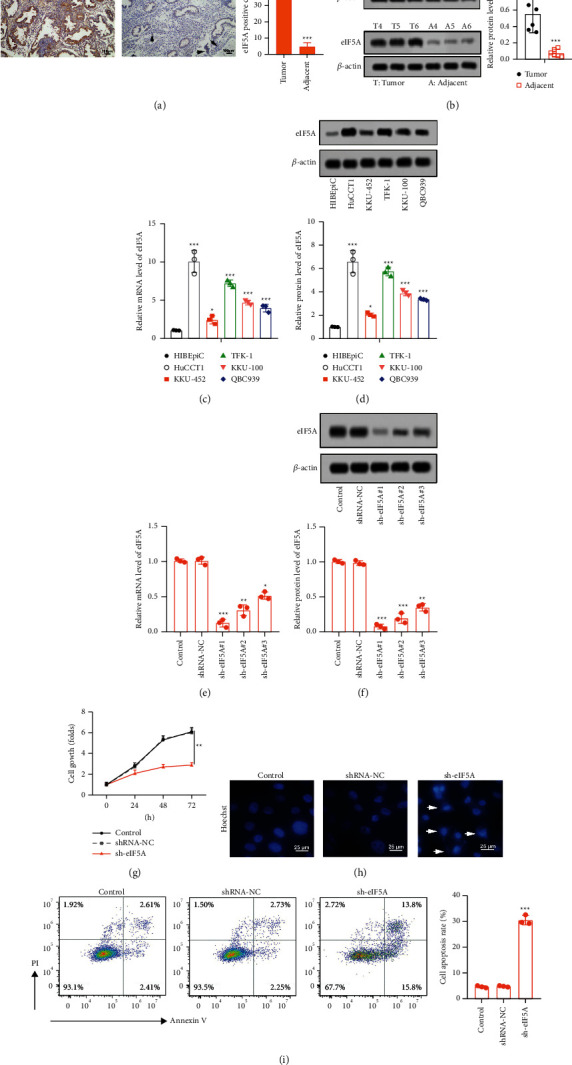
eIF5A was overexpressed in CCA tissues and cells, and silencing eIF5A suppressed cell growth. (a, b) eIF5A expression was assessed by immunohistochemistry and western blot. The nonneoplastic bile canaliculi in adjacent tissues have been pointed out by black arrows. (c, d) eIF5A expression in CCA cells was evaluated by RT-PCR and western blot. (e, f) After transfection with sh-eIF5A (^#^1, 2, 3) or shRNA-NC, the expression of eIF5A was detected by RT-PCR and western blot. (g) Cell viability was tested by CCK8 assay. (h, i) Cell apoptosis was evaluated by Hoechst 33258 standing, and flow cytometry. The morphological changes of apoptosis have been pointed put by arrows. ^*∗∗∗*^*p* < 0.001 vs. tumor group; ^*∗*^*p* < 0.05, ^*∗∗*^*p* < 0.01, and ^*∗∗∗*^*p* < 0.001 vs. HIBEpiC group; ^*∗*^*p* < 0.05 vs. control group, ^*∗∗*^*p* < 0.01 vs. control group, and ^*∗∗∗*^*p* < 0.001 vs. control group.

eIF5A expression in CCA tissues and cells was further measured. Similarly, eIF5A was highly expressed in CCA tissues and cells (Figures 3(a)–3)(d). HuCCT1 cells were selected for follow-up study. Next, the function of eIF5A in CCA cells was evaluated. eIF5A was downregulated by transfecting sh-eIF5A (^#^1, 2, 3) into HuCCT1 cells. The results indicated that eIF5A was significantly downregulated in sh-eIF5A groups (Figures 3(e) and 3)(f). Moreover, sh-eIF5A ^#^1 possessed most obvious interference effect, which is used in subsequent experiments. The results of CCK8 indicated that downregulation of eIF5A significantly repressed the activity of HuCCT1 cells at 48 h and 72 h (Figure 3)(g). Moreover, the results of Hoechst staining showed that sh-eIF5A increased the nuclear fragmentation and cell blebbing (Figure 3)(h). In addition, eIF5A silencing could promote HuCCT1 cell apoptosis, which was confirmed by flow cytometry assay (Figure 3)(i). The findings indicated that eIF5A might be an oncogene in CCA.

### 3.4. Downregulation of eIF5A Promoted Ferroptosis and Mitochondrial Apoptosis in CCA Cells

**Figure 4:**
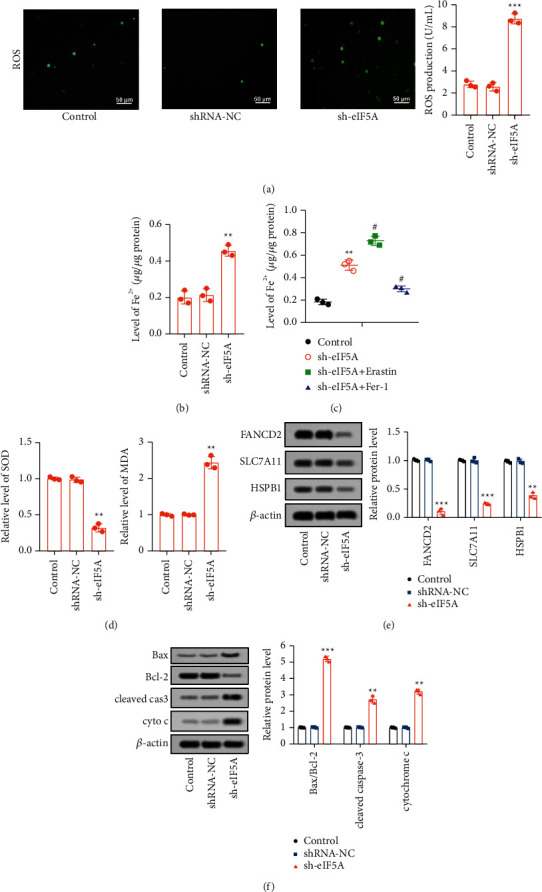
Downregulation of eIF5A enlarged ferroptosis by enhancing mitochondrial injury. (a) ROS level in CCA cells was assessed by DCFH-DA probe. (b–d) Fe2+, SOD, and MDA content were evaluated by according kits. (e) Ferroptosis markers (FANCD2, SCL7A11, and HSPB1) were measured by western blot. (f) Mitochondrial apoptosis proteins (Bcl-2, Bax, cleaved caspase-3, and cyto C) were detected by western blot. ^*∗∗*^*p* < 0.01 vs. control group, ^*∗∗∗*^*p* < 0.001 vs. control group, and ^#^*p* < 0.01 vs. sh-eIF5A.

The relationship of eIF5A and ferroptosis in CCA was explored. It was found that downregulation of eIF5A increased the level of intracellular ROS (Figure 4)(a). Moreover, eIF5A silencing enlarged the level of Fe^2+^ in CCA cells (Figure 4)(b), which enhanced erastin-induced ferroptosis and reduced fer-1-induced ferroptosis inhibition (Figure 4)(c). In addition, SOD activity was attenuated, but MDA levels were upregulated in eIF5A-silenced CCA cells (Figure 4)(d). Ferroptosis marker proteins FANCD2, SLC7A11, and HSPB1 were significantly restrained in CCA cells transfected with sh-eIF5A (Figure 4)(e). Besides, eIF5A silencing could amplify mitochondrial apoptosis, enhance Bax, cleaved caspase-3 and cytochrome C (cyto C), and reduce the expression of Bcl-2 (Figure 4)(f). These results indicated that inhibition of eIF5A enhanced CCA cells ferroptosis and mitochondrial apoptosis in CCA.

### 3.5. Upregulation of eIF5A Promoted the Growth of CCA Cells and Inhibited Ferroptosis of CCA Cells

**Figure 5:**
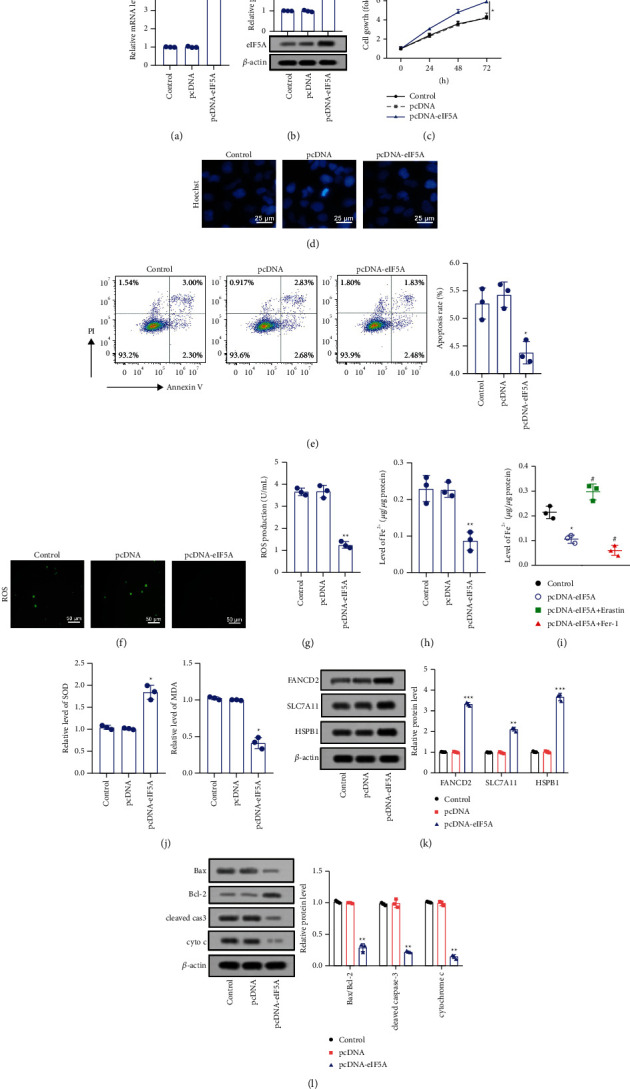
Upregulation of eIF5A increased cell viability and restrained ferroptosis by attenuating mitochondrial injury. (a, b) After transfection with pcDNA-eIF5A or pcDNA-NC, the expression of eIF5A was detected by RT-PCR and western blot. (c) Cell viability was tested by CCK8 assay. (d, e) Cell apoptosis was measured by Hoechst 33258 standing and flow cytometry. (f, g) ROS level in CCA cells was assessed by DCFH-DA probe. (h–j) Fe^2+^, SOD, and MDA content were evaluated by according kits. (k) Ferroptosis markers (FANCD2, SCL7A11, and HSPB1) were measured by western blot. (l) Mitochondrial apoptosis proteins (Bcl-2, Bax, cleaved caspase-3, and cyto C) were detected by western blot. ^*∗*^*p* < 0.05 vs. control group, ^*∗∗*^*p* < 0.01 vs. control group, ^*∗∗∗*^*p* < 0.001 vs. control group, and ^#^*p* < 0.01 vs. eIF5A group.

To further verify the function of eIF5A in CCA cells, PcDNA-eIF5A was transfected into KKU-452 cells, which induced the overexpression of eIF5A (Figures 5(a) and 5)(b). eIF5A could significantly increase the activity of KKU-452 cells at 48 h and 72 h (Figure 5)(c). The results of Hoechst staining indicated that eIF5A overexpression has no significant influence on the morphological changes of cells (Figure 5)(d). Apoptosis of KKU-452 cells was reduced by upregulation of eIF5A (Figure 5)(e). Moreover, ROS levels were significantly reduced in KKU-452 cells transfected with pcDNA-eIF5A (Figures 5(f) and 5)(g). Overexpression of eIF5A reduced Fe^2+^ levels (Figure 5)(h), which attenuated erastin-induced ferroptosis and enlarged Fer-1-induced ferroptosis (Figure 5)(i). The increased SOD activity and reduced MDA levels were found in KKU-452 cells of upregulation of eIF5A (Figure 5)(j). eIF5A significantly increased the expression of ferroptosis marker proteins FANCD2, SLC7A11, and HSPB1 (Figure 5)(k). In addition, eIF5A inhibited mitochondrial apoptosis through amplifying Bcl-2 and weakening Bax, cleaved caspase-3, and cyto C (Figure 5(l). The data suggested that upregulation of eIF5A restrains cell ferroptosis and apoptosis in CCA cells.

### 3.6. eIF5A Silencing Suppressed the Growth of CCA Xenografted Tumors

**Figure 6:**
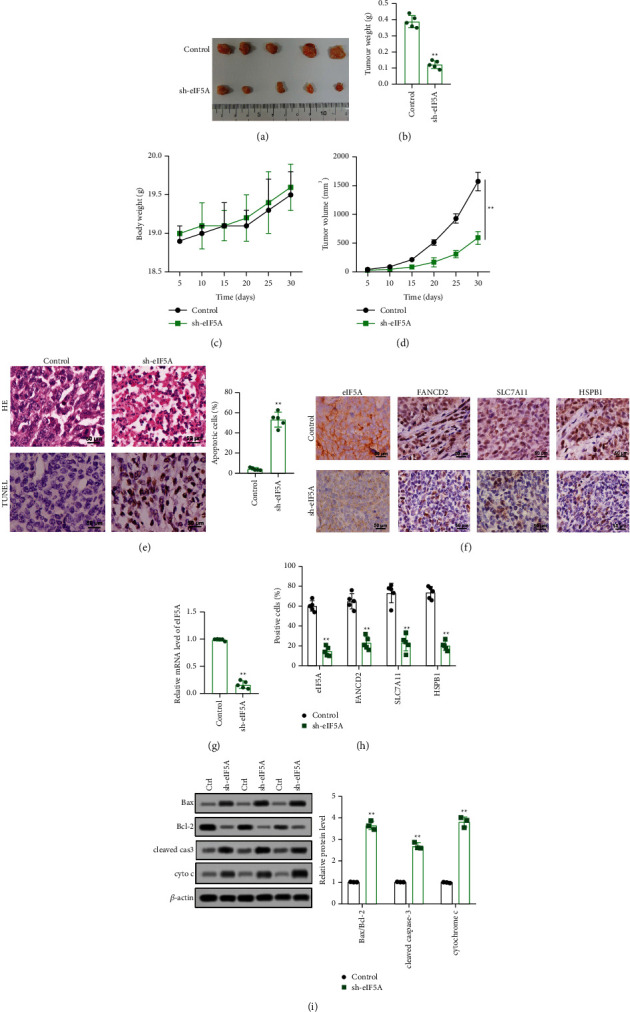
Silencing eIF5A suppressed the growth of xenografted tumors. The mice were divided into control group (*n* = 9) and sh-eIF5A group (*n* = 9). (a) Obtained xenografted tumors were photographed. (b) Obtained xenografted tumors were weighed. (c) The growth curve of mice body weight. (d) The growth curve of tumor volume. (e) Pathological changes and apoptosis were detected by HE staining and TUNEL. (f–h) The expression levels of eIF5A, FANCD2, SCL7A11, and HSPB1 were assessed by immunohistochemistry. (i) The expression levels of Bcl-2, Bax, cleaved caspase-3, and cyto C were assessed by western blot. ^*∗∗*^*p* < 0.01 vs. control group.

In order to explore the effect of eIF5A on CCA cells in vivo, a xenografted tumor model was constructed by subcutaneous injection of CCA cells in the back of nude mice. The results indicated that the blocking of eIF5A could reduce the size and weight of tumors (Figures 6(a) and 6)(b). The tumor growth curve showed that eIF5A silencing could restrain tumor growth (Figures 6(c) and 6)(d). Moreover, compared with control group, the tumor tissue was loose and necrotic tumor cells could be observed in sh-eIF5A group. Besides, downregulation of eIF5A promoted tumor cell apoptosis (Figures 6). Furthermore, the results of immunohistochemistry suggested that eIF5A was significantly reduced (Figures 6(f) and 6)(g), and the ferroptosis marker proteins (FANCD2, SLC7A11, and HSPb1) were significantly downregulated in the tumor tissues of sh-eIF5A group mice (Figures 6(f) and 6)(h). In addition, the downregulation of eIF5A increased the expression of Bax, cleaved caspase-3, and cyto C and reduced the expression of Bcl-2 in tumor tissues (Figure 6)(i). These results demonstrated that silencing of eIF5A repressed the growth of xenograft and enlarged cell apoptosis and ferroptosis.

## 4. Discussion

Targeted therapy is an attractive therapeutic approach for cancer [28, 29]. The screening of target genes is urgently required for the treatment of CCA. eIF5A is a ubiquitous protein in fungi, animals, and plants [30]. Studies have found that eIF5A is a newly discovered oncogene of the eIF5A family and demonstrates a key regulatory role in the pathogenesis of various cancers [31, 32]. The study reported that silencing of eIF5A-2 repressed cell migration and invasion in lung cancer [33]. eIF5A has negative correlation with survival rate in colorectal cancer patients compared with high expression of eIF5A gene [34]. eIF5A2 modulated the metastasis and invasion of hepatocellular carcinoma [35]. However, whether eIF5A was abnormally expressed in CCA and affected the properties of CCA is still unclear. In our study, both bioinformatics analysis and experimental data demonstrated that eIF5A might be an oncogene in CCA. Similarly, our findings indicated that eIF5A was highly expressed in CCA, and eIF5A was positively correlated with pT and pTNM stages and negatively correlated with overall survival. eIF5A expression, age, and pT stage were independent risk factors for the patients with CCA. Moreover, eIF5A increased the activity of CCA cells and inhibited ferroptosis and mitochondrial apoptosis.

In essence, ferroptosis is kind of cell death way due to the imbalance of intracellular lipid oxide metabolism caused by iron overload and the production of ROS [9]. Ferroptosis was first found in tumor cells studying RAS-mutations [36, 37], which was involved in the progression of multiple tumors [38, 39]. Hasegawa et al. reported that inhibition of MUCI-C/System Xc pathway could inhibit three-negative breast cancer progression by causing ferroptosis [40]. Chang et al. found that BAY 11–7085 (I*κ*B*α* inhibitor) triggered ferroptosis through Nrf2-SLC7A11-HO-1 pathway, thus showing the effect of anti-head and neck cancer [41]. The possible mechanism was that BAY 11–7085 aggregated HO-1 to the nucleus and mitochondria, promoted mitochondrial autophagy, and further induced ferroptosis [41]. Among the known ferroptosis regulatory proteins, cysteinyl transfer ribonucleic acid synthase (CARS) and transferrin receptor 1 (TER1) showed positive feedback regulation, while cystine glutamate transporter (SLC7A11), heat shock protein B1 (HSPB1), FANCD2, and glutathione peroxidase (GPX4) showed negative feedback regulation [42, 43]. The study has reported that the overexpression of SLC7A11 promoted tumor growth by partially inhibiting ferroptosis [44]. Knockout of HSPB1 could enhance erastin-induced ferroptosis, but upregulation of HSPB1 could inhibit this effect in cancer cells [45]. Overexpression of FANCD2, as an ferroptosis-related suppressor gene, might be an important prognostic indicator in clear cell renal cell carcinoma [46]. Similarly, SLC7A11, HSPB1, and FANCD2 were abnormally upregulated in CCA, which demonstrated that ferroptosis might participate in regulating the progression of CCA. Moreover, the downregulation of eIF5A could significantly inhibit the expression of SLC7A11, HSPB1, and FANCD2, thus promoting ferroptosis in CCA cells.

The downregulation of eIF5A increased the production of ROS by inducing mitochondrial damage, which may be an important factor in inducing cancer ferroptosis [47, 48]. Ferroptosis inducers could connect with membrane porin 2 and membrane porin 3 on the outer membrane of mitochondria, change the permeability of mitochondrial membrane, reduce the sensitivity of channels to iron ions, limit the outflow of substances in mitochondria, cause mitochondrial dysfunction and release of a large number of oxidizing substances, and finally lead to ferroptosis [49]. We hypothesized that eIF5A might inhibit the occurrence of ferroptosis in CCA by reducing mitochondrial damage. Interestingly, our study suggested that silencing of eIF5A increased ROS and Fe^2+^ levels and enlarged mitochondrial apoptosis, which might further promote ferroptosis. Finally, the results of in vivo experiments further verified that eIF5A promoted the growth of CCA cells and restrained ferroptosis. However, the limitations of our study were that we did not confirm whether eIF5A affected the growth of CCA through other signaling pathways and whether eIF5A affected other phenotypes of CCA (such as cell migration, invasion, and stem cell characteristics). Those need be further explored in the future.

## 5. Conclusion

Clinical and experimental data indicated that eIF5A was overexpressed in CCA tissues and cells, and high expression of eIF5A showed poor survival and advanced disease stage in patients with CCA, indicating that eIF5A might be a potential prognostic indicator of CCA. The results of cell function experiment showed that eIF5A increased the activity of CCA cells and inhibited ferroptosis by attenuating mitochondrial dysfunction. These results suggested that target inhibition of eIF5A might be an effective treatment strategy for CCA.

## Figures and Tables

**Table 1:** Correlation between eIF5A expression and the clinical pathological features of cholangiocarcinoma patients.

	Charar	eIF5A expression		*P* value
		High	Low	
Status	Alive	10	21	0.022^*∗*^
	Dead	18	7	

Age	Mean (SD)	63.4 (12.9)	62.6 (13.1)	0.849
	Median (MIN, MAX)	65.5 (31, 82)	67 (29, 81)	

Gender	Female	9	11	0.737
	Male	19	17	

Race	Asian	11	12	0.828
	White	17	14	
	Black		2	

pT_stage	T1	6	21	0.002^*∗∗*^
	T2	9	5	
	T3	13	2	

pN_stage	N0	12	14	0.758
	N1	13	12	
	NX	3	2	

pM_stage	M0	11	15	0.066
	M1	15	12	
	MX	2	1	

pTNM_stage	I	8	20	0.004^*∗∗*^
	II	4	5	
	III	14	3	
	IV	2		

new_tumor_event_type	Primary	9	7	0.915
	Recurrence	19	21	

^*∗*^ *p* < 0.05, ^*∗∗*^*p* < 0.01 vs. high eIF5A expression group.

## Data Availability

The datasets used and/or analyzed during the current study are available from the corresponding author on reasonable request.

## Supplementary Materials

Supplementary Materials Supplementary Figure 1: the relation of eIF5A expression and immune cell infiltration was researched through TIMER database.
