# Supplementary material for: Eukaryotic Translation Initiation Factor 5A Independently Predicts Poor Prognosis of Cholangiocarcinoma Patients and Regulates the Ferroptosis and Mitochondrial Apoptosis
Source: J Oncol. 2022 Jul 15;2022:4250531. doi: 10.1155/2022/4250531 (PMC9307416; doi:10.1155/2022/4250531)
Supplement: Supplementary Materials — Supplementary Figure 1: the relation of eIF5A expression and immune cell infiltration was researched through TIMER database. [file 4250531.f1.pdf]

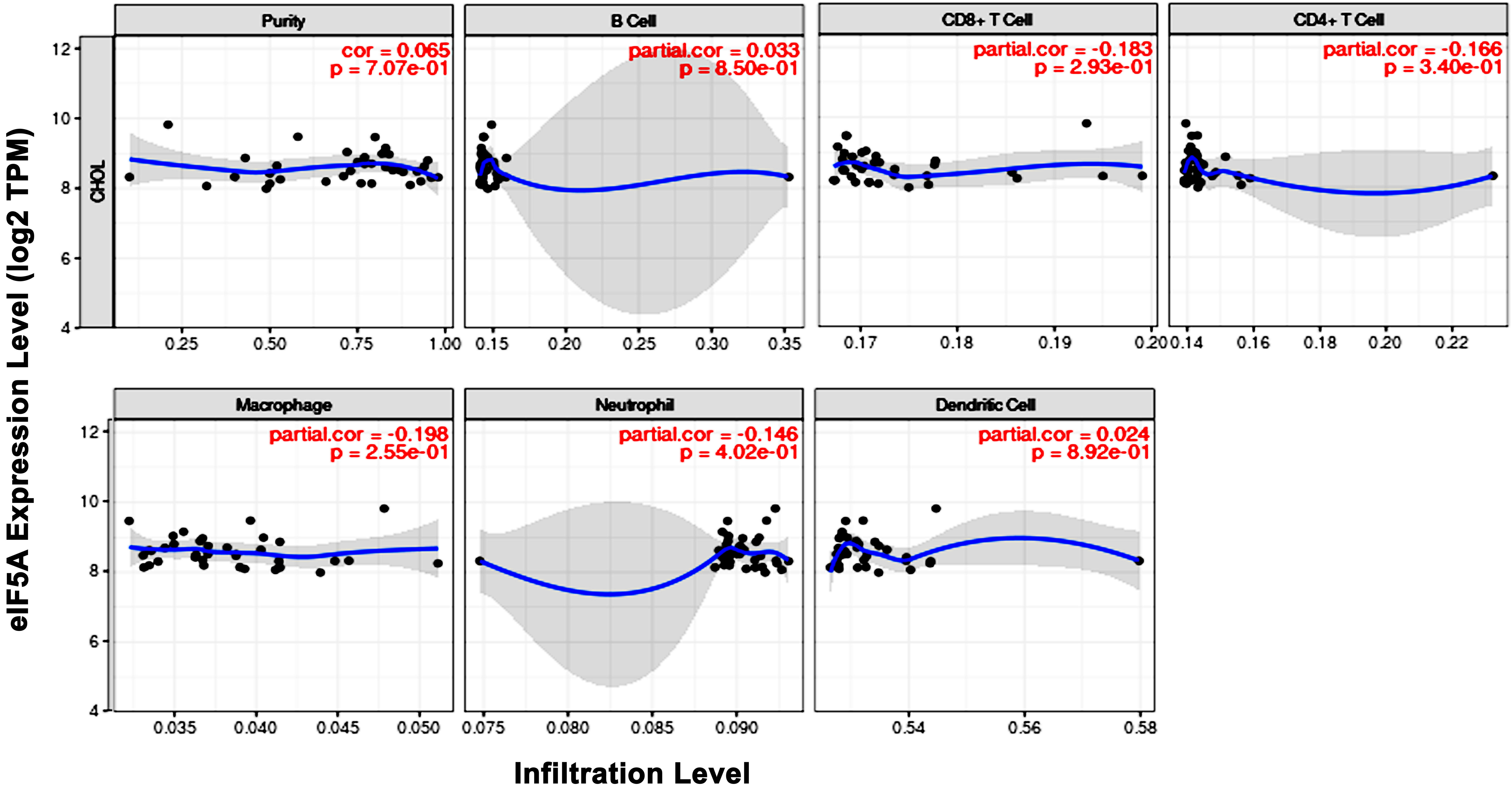

Purity
cor = 0.065
p = 7.07e-01
B Cell
partial.cor = 0.033
p = 8.50e-01
CD8+ T Cell
partial.cor = -0.183
p = 2.93e-01
CD4+ T Cell
partial.cor = -0.166
p = 3.40e-01
Macrophage
partial.cor = -0.198
p = 2.55e-01
Neutrophil
partial.cor = -0.146
p = 4.02e-01
Dendritic Cell
partial.cor = 0.024
p = 8.92e-01
CHOL
eIF5A Expression Level (log2 TPM)
Infiltration Level
